# Association between serum carotenoid levels and gallstones in adults: a cross-sectional study using the National Health and Nutrition Examination Survey

**DOI:** 10.3389/fnut.2025.1601010

**Published:** 2025-07-03

**Authors:** Weirui Ren, Chuang Zhang, Jie Yin, Jingyi Ren, Hongzhao Song, Xiaoya Wang, Junmin Wang

**Affiliations:** ^1^Department of Gastroenterology, Hebei Medical University Third Hospital, Shijiazhuang, China; ^2^Department of Clinical Medicine, Shijiazhuang Medical College, Shijiazhuang, China; ^3^Department of Gastroenterology, The Second Hospital of Hebei Medical University, Shijiazhuang, China; ^4^Department of Graduate College, Hebei Medical University, Shijiazhuang, China

**Keywords:** serum carotenoids, gallstones, NHANES, BMI, cross-sectional study, antioxidants, inflammation, mediation analysis

## Abstract

**Background:**

Carotenoids, known for their antioxidant properties and potential health benefits, have attracted considerable attention. Nonetheless, the association between carotenoid levels and gallstone prevalence has not been adequately explored. This study aimed to investigate the association between serum carotenoid levels and gallstone risk in an adult population.

**Methods:**

This cross-sectional study utilized the 2017–2018 NHANES data. Multivariate logistic regression analyses were performed to assess the association between serum carotenoid levels and gallstone prevalence. Subgroup and interaction analyses were conducted to confirm these findings. Additionally, generalized additive model (GAM) regression combined with smooth curve fitting techniques was utilized to clarify potential non-linear associations, and a mediation analysis was conducted to identify possible mediators in the association between serum carotenoid levels and gallstones.

**Results:**

This study included 3,809 participants aged ≥20 years, among whom 412 had gallstones. After adjusting for confounders (Model 3), serum carotenoid levels were inversely associated with gallstone prevalence. The participants in the highest quartile of total carotenoid levels had a 48% lower gallstone risk than those in the lowest quartile (Q4: odds ratio [OR] = 0.52, *p* = 0.0005). Individual carotenoids showed similar trends: *α*-carotene (49% lower risk, OR = 0.51, *p* = 0.0010), α-cryptoxanthin (54% lower risk, Q4: OR = 0.46, *p* < 0.0001), β-carotene (47% lower risk, Q4: OR = 0.53, *p* = 0.0010), β-cryptoxanthin (42% lower risk, Q4: OR = 0.58, *p* = 0.0061), lutein/zeaxanthin (44% lower risk, Q4: OR = 0.56, *p* = 0.0025), and lycopene (30% lower risk, Q4: OR = 0.70, *p* = 0.0441). GAM analysis detected non-linear associations between carotenoids and gallstone risk. The subgroup and interaction analyses confirmed these results. Mediation analysis revealed that body mass index (BMI) accounted for 16.7% of the total effect.

**Conclusion:**

Observational data demonstrated inverse associations between serum carotenoid levels and gallstone prevalence, with BMI mediating 16.7% of the total effect. These findings suggest that maintaining high serum carotenoid levels may reduce the gallstone risk. Future studies should explore the protective mechanisms of carotenoids and validate their causal relationships using longitudinal studies.

## 1 Introduction

Gallstones, a common digestive disease worldwide, affect approximately 10–20% of the adult population, imposing a heavy medical and economic burden globally (1, 2). Regional variations in prevalence exist, with rates of 10–15% in the United States, 9–21% in Europe, and 10% in Asian populations, all showing a global upward trend (3, 4). Gallstone formation is associated with various genetic and environmental factors including race, sex, age, family history, obesity, diet, lifestyle, and medication (5, 6). Although most gallstone patients remain asymptomatic throughout their lives, approximately 10% eventually develop clinical manifestations such as significant abdominal discomfort and digestive dysfunction (7). Additionally, 1–2% of gallstone patients develop complications, such as cholecystitis, acute suppurative cholangitis, and pancreatitis, which seriously affect their health and quality of life (5, 8, 9). Furthermore, gallstone disease is associated with an increased risk of hepatobiliary and pancreatic malignancies and is an independent risk factor for gallbladder cancer-related mortality (10, 11). Given its status as a major global public health challenge and its potential serious consequences, further exploration of the underlying causes of gallstones and preventive measures are crucial to reduce the global public health burden associated with this disease.

Gallstone development is a complex, multifactorial phenomenon in which oxidative stress and inflammatory processes play important role (12). Studies have shown that the occurrence of gallstones is associated with elevated oxidative stress levels and decreased antioxidant capacity, and the levels of oxidative stress markers (such as malondialdehyde and superoxide dismutase) in patients with gallstones (13, 14). Oxidative stress damages the gallbladder mucosal epithelial cells and triggers lipid peroxidation by generating reactive oxygen species, thereby instigating inflammatory cascades. Activation of inflammatory mediators stimulates macrophages, resulting in the release of pro-inflammatory substances and the establishment of a chronic inflammatory milieu (15–17). This interaction leads to bile cholesterol supersaturation and irregular bilirubin metabolism, ultimately promoting gallstone formation of gallstones (18, 19).

Carotenoids, which are prevalent natural antioxidants, encompass a range of compounds including *α*-carotene, α-cryptoxanthin, β-carotene, β-cryptoxanthin, lutein/zeaxanthin, and lycopene, collectively representing over 95% of carotenoids found in human serum (20). These serum carotenoids have significant biological functions and exhibit antioxidant, anti-inflammatory, and anticancer properties that are essential for mitigating various health concerns, particularly cardiovascular diseases, metabolic syndromes, and other chronic disorders (21–23). Under normal circumstances, there is a good correlation between the dietary intake of certain fruits and vegetables and the concentration of carotenoids in the blood. However, compared to the intake of dietary carotenoids, serum carotenoids show a stronger and more linear negative correlation with the occurrence of diseases (24). Given their bioactive potential, carotenoids may contribute to gallstone prevention by alleviating oxidative stress and inflammation. However, the association between serum carotenoid levels and gallstone prevalence in large adult cohorts remains inadequately explored. This study aimed to analyze National Health and Nutrition Examination Survey (NHANES) data to examine the association between serum carotenoid levels and gallstone risk among adults in the United States. We hypothesized that higher serum carotenoid levels would correlate with lower gallstone risk. To test this, we used multiple methods including multivariate logistic regression, generalized additive model (GAM), and mediation analysis. Our findings are expected to offer new insights and strategies for gallstone prevention and intervention, supporting clinical and public health decision-making.

## 2 Materials and methods

### 2.1 Data sources and study population

**Figure 1:**
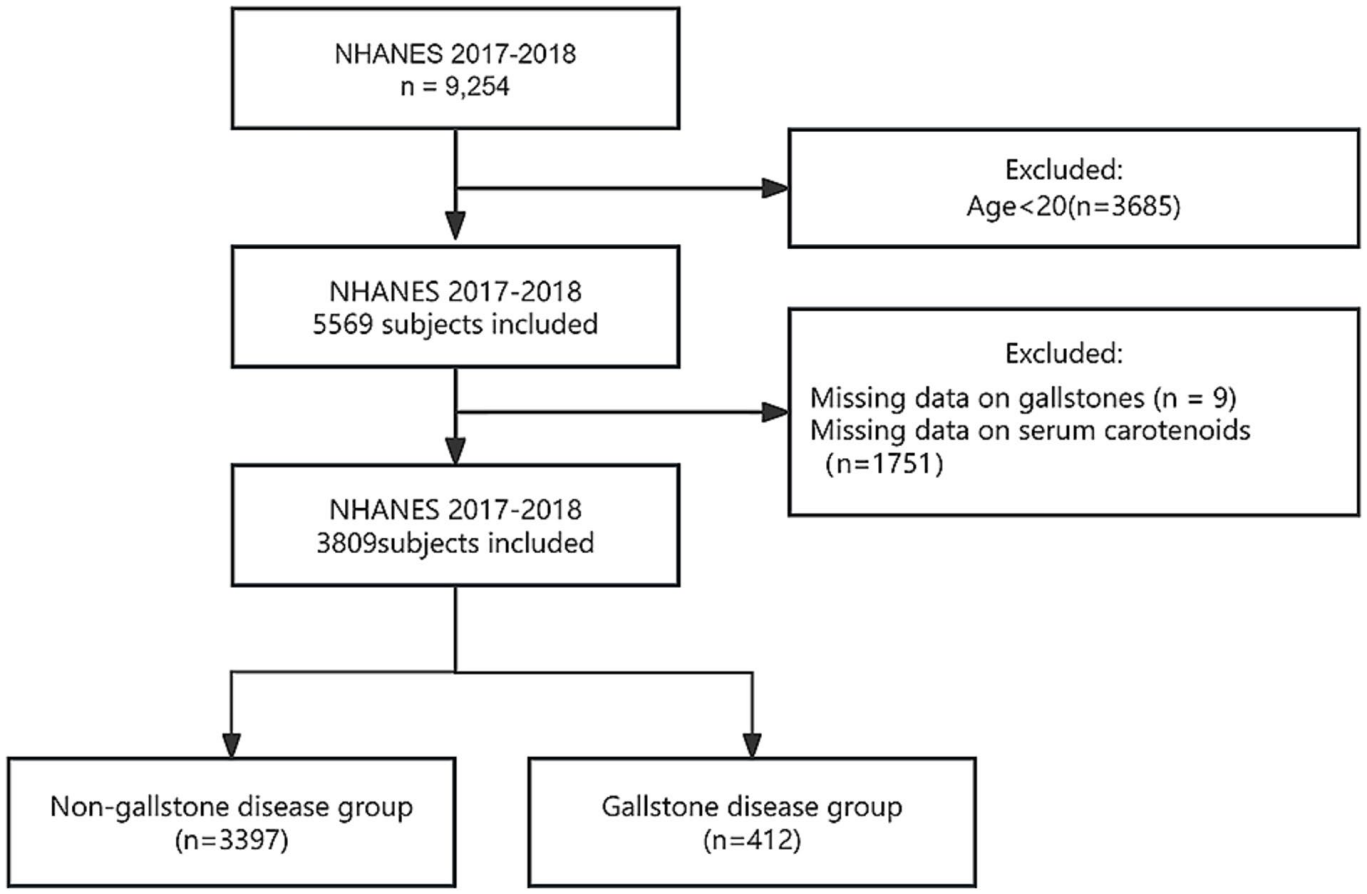
Flowchart of participant screening.

This study used data derived from the NHANES. Administered by the National Center for Health Statistics (NCHS), the NHANES uses a stratified multistage sampling framework to evaluate the nutritional and health profiles of adults and children across the United States (25). The research methodology was approved by the NCHS Institutional Review Board, and all participants provided written informed consent prior to their participation. Given that the data utilized in this study originated exclusively from the NHANES public database, individual patient identifiers were substituted with identification codes, thereby eliminating the need for ethical approval from the authors’ affiliated institutions (26). The dataset for this study was collected during the 2017–2018 NHANES cycle and presented extensive data on serum carotenoid levels and gallstone prevalence. Initially, the study included 9,254 participants. The exclusion criteria were as follows: (1) individuals younger than 20 years (*n* = 3,685), (2) participants lacking gallstone data (*n* = 9), and (3) participants with missing carotenoid data (*n* = 1,751). Following the application of these exclusion criteria, the final dataset comprised 3,809 participants, including 412 individuals diagnosed with gallstones and 3,397 individuals without gallstones (Figure 1).

### 2.2 Serum carotenoids measurement

This study focuses on essential serum carotenoids, which encompass *α*-carotene, α-cryptoxanthin, *β*-carotene, β-cryptoxanthin, lutein/zeaxanthin, and lycopene. The serum concentration of β-carotene is derived by aggregating the amounts of trans-β-carotene and cis-β-carotene. The total serum carotenoid (TSC) level was determined by summing the levels of six primary carotenoids. The NHANES database utilizes photodiode array detection (PDAD) to quantify the serum levels of these carotenoids (27). To ensure precision of the measurements, the research team formulated comprehensive laboratory procedures and quality control measures documented in the NHANES Laboratory/Medical Technologists Procedures Manual (28).

### 2.3 Definition of gallstones

Gallstones were identified as the outcome variables in the context of this study. We assessed patients with gallstones through self-reported interviews using the Medical Condition Questionnaire (MCQ) from the NHANES (2017–2018) dataset. Participants were asked the question, “Has a doctor or other health professional ever informed you that you have gallstones?” Those responding “yes” were categorized as having gallstones, whereas those denying this diagnosis were classified as not having gallstones.

### 2.4 Assessment of covariates

To improve the precision and thoroughness of the analysis examining the association between serum carotenoid levels and gallstones in adults, the research team recognized several covariates based on clinical expertise and prior studies (29–31). The significant covariates included demographic factors, comorbid conditions, and other pertinent variables, as outlined in Table 1. In the analysis, the poverty income ratio (PIR) was stratified into three categories: <1.3, 1.3–3.5, and >3.5. Recorded comorbidities included hypertension, diabetes, coronary heart disease (CHD), thyroid disorders, stroke, and cancer or malignancy. Hypertension is defined by satisfying at least one of the following criteria: (i) average systolic blood pressure ≥140 mmHg and/or diastolic blood pressure ≥90 mmHg, measured three times on the same day; (ii) current utilization of antihypertensive medication; (iii) self-reported hypertension through the questionnaire. Diabetes is similarly characterized by meeting one of these criteria: (i) hemoglobin A1c (HbA1c) level ≥6.5%; (ii) fasting blood glucose (FBG) level ≥126 mg/dL; (iii) self-reported diabetes diagnosis or current use of insulin or antidiabetic medications. Definitions of CHD and other related complications were based on responses to the medical history questionnaire. Smoking history was defined as having smoked a minimum of 100 cigarettes over a lifetime. Participants who consumed at least 12 alcoholic beverages per year were classified as alcohol drinkers.

**Table 1:** Covariates extracted from the 2017–2018 NHANES.

Items	Composition
Demographic variables	Age, gender, ethnicity, education level, marital status, PIR
Comorbidities	Hypertension, diabetes, CHD, thyroid problem, stroke, thyroid problem, cancer/malignancy
Others	BMI, moderate recreational activities, alcohol consumption, smoking status

PIR, poverty income ratio; CHD, coronary heart disease; BMI, body mass index.

### 2.5 Statistical analysis

The statistical evaluation conducted in this study adhered to the guidelines set forth by the NHANES and incorporated appropriate sampling weights. Categorical variables were represented as unweighted frequencies and weighted percentages, whereas continuous variables were characterized by weighted medians and interquartile ranges because of their non-normal distribution. Chi-square and Mann–Whitney U tests were used to compare categorical and continuous variables. To examine the association between serum carotenoid levels and the presence of gallstones, carotenoid levels were stratified into quartiles, with the lowest quartile serving as the reference group. Three distinct logistic regression models were developed: Model 1 was unadjusted; Model 2 was controlled for variables such as age, sex, ethnicity, marital status, educational level, and PIR; and Model 3 was additionally adjusted for the covariates included in Model 2, as well as for body mass index (BMI), smoking, alcohol consumption, physical activity, diabetes, hypertension, CHD, stroke, thyroid disorders, and cancer/malignancy. We computed *p*-values for trends to determine whether the odds ratio (OR) for gallstones fluctuated with ascending carotenoid quartiles. Moreover, GAM regression combined with smooth curve fitting was utilized to assess potential non-linear associations, with effective degrees of freedom (EDF) serving as an indicator of curvature. An EDF value of 1 reflects a linear association, whereas values exceeding 1 indicate a more intricate association. Spearman’s correlation coefficients were calculated to evaluate the associations between serum carotenoids, and weighted quantile sum (WQS) regression was applied to investigate the cumulative effects of the six carotenoids on gallstone occurrence. Further analyses involving subgroups and interaction tests were performed to discern consistency across various subpopulations and to scrutinize the influences and interactions of covariates, such as age, sex, ethnicity, educational level, PIR, BMI, diabetes, and CHD. Potential mediators that might influence the association between serum carotenoids and gallstones were also explored, including liver function markers (alanine aminotransferase [ALT], aspartate aminotransferase [AST], gamma-glutamyl transferase [GGT], total bilirubin), uric acid, inflammatory markers (C-reactive protein [CRP], neutrophil count, ferritin), obesity metrics (BMI), lipid profiles (triglycerides, total cholesterol), along with diabetes and insulin resistance indicators (HbA1c, FBG, fasting serum insulin). All statistical analyses were performed using R software version 4.3.2^1^ and EmpowerStats software version 4.2^2^. Statistical significance was set at *p* < 0.05.

## 3 Results

### 3.1 Baseline characteristics of study participants

Table 2 provides a comprehensive overview of participants’ baseline characteristics. A total of 3,809 individuals were analyzed, of which 412 (10.8%) were diagnosed with gallstones. In comparison to those without gallstones, individuals with gallstones were significantly older (mean age of 59.00 years versus 47.00 years, *p* < 0.0001) and exhibited a greater proportion of females (74.76% compared to 49.29%, *p* < 0.0010). Examination of the racial distribution within the gallstone cohort revealed that 7.89% were Hispanic Americans, 70.31% were non-Hispanic White, 6.92% were non-Hispanic Black, and 14.88% were from other racial backgrounds (*p* = 0.0488). Patients with gallstones displayed markedly elevated rates of hypertension (60.12% vs. 37.57%, *p* < 0.0001), diabetes (27.23% vs. 14.94%, *p* = 0.0001), CHD (8.44% vs. 3.86%, *p* = 0.0023), thyroid disorders (23.27% vs. 10.90%, *p* < 0.0010), and stroke (6.92% vs. 3.04%, *p* = 0.0010) compared to their counterparts without gallstones. Additionally, the prevalence of malignancies was significantly higher among patients with gallstones (18.35%, 79/412) than among those without gallstones (9.82%, 310/3,397, *p* < 0.0010). With respect to BMI, patients with gallstones had a significantly higher proportion (56.45%) with BMI > 30 kg/m^2^ than patients without gallstones (39.48%) (*p* < 0.0001). Biochemical markers indicated that patients with gallstones had significantly elevated FBG level (6.05 vs. 5.66, *p* = 0.0166), triglyceride level (1.25 vs. 1.02, *p* = 0.0654), white blood cell count (7.70 vs. 7.00, *p* = 0.0055), and neutrophil count (4.50 vs. 4.10, *p* = 0.0009). Moreover, serum carotenoid levels were significantly lower in individuals with gallstones than in those without (*p* < 0.05). No significant disparities were noted between gallstone and non-gallstone patients regarding education levels, smoking habits, alcohol consumption, moderate leisure activities, PIR, and total bilirubin, total cholesterol, ferritin, ALT, AST, GGT, lactate dehydrogenase, TB, and uric acid levels (*p* > 0.05).

**Table 2:** Baseline characteristics of the participants.

Characteristics	Overall	Gallstones	No gallstones	*P*-value
Unweighted number	3,809	412	3,397	
Weighted number	171,480,708	19,244,861	152,235,847	
Age (IQR)	48.00 (33.00,62.00)	59.00 (45.00, 69.00)	47.00 (33.00, 61.00)	<0.0001
Gender, *n* (weighted %)				<0.0010
Male	1842 (47.85%)	115 (25.24%)	1727 (50.71%)	
Female	1967 (52.15%)	297 (74.76%)	1,670 (49.29%)	
Ethnicity, *n* (weighted %)				0.0488
Mexican American	495 (8.58%)	60 (7.89%)	435 (8.67%)	
Non-Hispanic White	1,327 (62.71%)	189 (70.31%)	1,138 (61.75%)	
Non-Hispanic Black	906 (11.42%)	71 (6.92%)	835 (11.98%)	
Other Races	1,081 (17.30%)	92 (14.88%)	989 (17.60%)	
Education level, *n* (weighted %)				0.8444
<High school	756 (11.26%)	78 (10.99%)	678 (11.30%)	
High school	900 (27.97%)	103 (29.52%)	797 (27.77%)	
>High school	2,147 (60.77%)	231 (59.49%)	1916 (60.93%)	
Marital status, *n* (weighted %)				0.0069
Married/Living with Partner	2,274 (63.66%)	246 (59.90%)	2028 (64.14%)	
Widowed/Divorced/Separated	879 (18.85%)	122 (27.26%)	757 (17.78%)	
Never married	654 (17.49%)	44 (12.83%)	610 (18.08%)	
Smoking, *n* (weighted %)	1,605 (42.02%)	192 (43.88%)	1,413 (41.79%)	0.6533
Alcohol drinking, *n* (weighted %)	3,195 (92.65%)	346 (92.44%)	2,849 (92.68%)	0.8963
Moderate recreational activities, n (weighted %)	1,558 (47.31%)	157 (47.57%)	1,401 (47.28%)	0.8903
Hypertension, *n* (weighted %)	1760 (40.10%)	251 (60.12%)	1,509 (37.57%)	<0.0001
Diabetes, *n* (weighted %)	845 (16.32%)	142 (27.23%)	703 (14.94%)	0.0001
CHD, *n* (weighted %)	184 (4.37%)	39 (8.44%)	145 (3.86%)	0.0023
Stroke, *n* (weighted %)	186 (3.48%)	38 (6.92%)	148 (3.04%)	0.0010
Thyroid problem, *n* (weighted %)	458 (12.29%)	90 (23.27%)	368 (10.90%)	<0.0010
Cnace/Malignant, *n* (weighted %)	389 (10.77%)	79 (18.35%)	310 (9.82%)	<0.0010
BMI, kg/m^2^, *n* (weighted %)				<0.0001
<=25	974 (26.28%)	49 (14.45%)	925 (27.77%)	
>25, <=30	1,237 (32.35%)	112 (29.09%)	1,125 (32.76%)	
>30	1,545 (41.37%)	240 (56.45%)	1,305 (39.48%)	
PIR, *n* (weighted %)				0.1002
<=1.3	942 (19.69%)	87 (18.80%)	855 (19.81%)	
>1.3, <=3.5	1,369 (35.90%)	169 (43.54%)	1,200 (34.95%)	
>3.5	1,017 (44.41%)	107 (37.66%)	910 (45.25%)	
α-carotene, μg/dL (IQR)	2.90 (1.49, 5.81)	2.54 (1.21, 5.02)	2.96 (1.53, 5.89)	0.0104
α-cryptoxanthin, μg/dL (IQR)	2.29 (1.63, 3.24)	1.79 (1.29, 2.44)	2.38 (1.70, 3.30)	<0.0001
β-carotene, μg/dL (IQR)	13.99 (7.74, 24.83)	12.70 (7.38, 22.52)	14.35 (7.82, 25.23)	0.0010
β-cryptoxanthin, μg/dL (IQR)	6.36 (3.99, 10.50)	4.82 (3.26, 7.91)	6.56 (4.09, 10.80)	0.0011
Lutein/zeaxanthin, μg/dL (IQR)	16.10 (11.20, 24.10)	14.30 (9.55, 22.90)	16.40 (11.50, 24.30)	0.0361
Lycopene, μg/dL (IQR)	37.90 (26.60, 50.70)	32.60 (22.90, 43.60)	38.50 (27.10, 51.30)	0.0009
TSC, μg/dL (IQR)	87.34 (62.66, 117.35)	74.91 (54.77, 101.04)	88.88 (64.03, 118.81)	0.0001
TG, mmol/L (IQR)	1.05 (0.68, 1.59)	1.25 (0.79, 1.81)	1.02 (0.67, 1.56)	0.0654
TC, mmol/L (IQR)	4.84 (4.19, 5.56)	4.78 (4.16, 5.59)	4.86 (4.19, 5.56)	0.7226
WBC, 1000 cells/μL (IQR)	7.00 (5.80, 8.60)	7.70 (6.30, 9.20)	7.00 (5.70, 8.50)	0.0055
NEU, 1000 cells/μL (IQR)	4.10 (3.10, 5.20)	4.50 (3.50, 5.60)	4.10 (3.10, 5.20)	0.0009
FBG, mmol/L (IQR)	5.72 (5.33, 6.22)	6.05 (5.55, 6.61)	5.66 (5.33, 6.11)	0.0166
FI, μU/mL (IQR)	9.15 (6.04, 15.40)	13.89 (8.54, 23.08)	8.76 (5.78, 14.32)	0.0024
HbA1c, % (IQR)	5.50 (5.20, 5.80)	5.70 (5.40, 6.00)	5.50 (5.20, 5.80)	0.0009
CRP, mg/L (IQR)	1.88 (0.86, 4.35)	2.72 (1.11, 6.98)	1.83 (0.84, 4.06)	0.0136
FER, ng/mL (IQR)	104.00 (51.00, 189.00)	109.00 (51.90, 166.00)	104.00 (51.00, 191.00)	0.7277
ALT, U/L (IQR)	18.00 (14.00, 26.00)	19.00 (14.00, 26.00)	18.00 (13.00, 26.00)	0.3720
AST, U/L (IQR)	19.00 (16.00, 24.00)	18.00 (15.00, 22.00)	19.00 (16.00, 24.00)	0.5461
GGT, IU/L (IQR)	20.00 (14.00, 31.00)	21.00 (15.00, 29.00)	20.00 (14.00, 32.00)	0.0567
LDH, IU/L (IQR)	155.00 (137.00, 173.00)	159.00 (139.00, 181.00)	154.00 (136.00, 172.00)	0.0734
TB, μmol/L (IQR)	6.84 (5.13, 10.26)	6.84 (5.13, 8.55)	6.84 (5.13, 10.26)	0.6682
UA, mg/dL (IQR)	5.30 (4.40, 6.30)	5.50 (4.50, 6.30)	5.30 (4.40, 6.30)	0.2652

Categorical variables were presented using unweighted frequencies and weighted percentages, whereas continuous variables were described using weighted medians and interquartile ranges owing to non-normal distribution.

CHD, coronary heart disease; PIR, poverty income ratio; CHD, coronary heart disease; BMI, body mass index; IQR, interquartile range; TSC, total serum carotenoid; TG, triglycerides; TC, total cholesterol; WBC, white blood cells; NEU, neutrophils; FBG, fasting blood glucose; FI, fasting insulin; HbA1c, hemoglobin A1c; CRP, C-reactive protein; FER, ferritin; ALT, alanine aminotransferase; AST, aspartate Aminotransferase; GGT, gamma-glutamyl transferase; LDH, lactate dehydrogenase; TB, total bilirubin; UA, uric acid.

### 3.2 The association between serum carotenoids and gallstones in adults

Table 3 summarizes the results of the association analysis between serum carotenoid levels and gallstone prevalence. After adjusting for confounding variables in Model 3, a significant negative correlation was identified between serum carotenoid levels and gallstone prevalence. Specifically, total carotenoid levels were negatively associated with gallstone prevalence, with ORs of 0.51 (*p* = 0.0002) for Q3 and 0.52 (*p* = 0.0005) for Q4, indicating a 48% reduction in gallstone prevalence among individuals in the highest quartile. For *α*-carotene, the OR values were 0.72 (*p* = 0.0446) for Q2, 0.54 (*p* = 0.0008) for Q3, and 0.51 (*p* = 0.0010) for Q4, demonstrating a negative correlation with gallstone prevalence. For α-cryptoxanthin, the OR values were 0.61 (*p* = 0.0015) for Q2, 0.44 (*p* < 0.0001) for Q3, and 0.46 (*p* < 0.0001), also showing a negative correlation with gallstone prevalence. Similarly, β-carotene exhibited OR values of 0.71 (*p* = 0.0365) for Q2, 0.46 (*p* < 0.0001) for Q3, and 0.53 (*p* = 0.0010) for Q4, further confirming this negative association. Additionally, β-cryptoxanthin displayed negative correlations, with OR values of 0.70 (*p* = 0.0290) for Q2, 0.65 (*p* = 0.0174) for Q3, and 0.58 (*p* = 0.0061) for Q4. Lutein/zeaxanthin also showed a significant negative correlation with Q3 (OR = 0.64, *p* = 0.0119) and Q4 (OR = 0.56, *p* = 0.0025). Furthermore, Lycopene levels were negatively correlated with gallstone prevalence in Q3 (OR = 0.53, *p* = 0.0005) and Q4 (OR = 0.70, *p* = 0.0441). Trend tests further supported the existence of a significant negative correlation between serum carotenoid levels and gallstone prevalence.

**Table 3:** Multivariate-adjusted association between serum carotenoid quartiles and gallstone prevalence.

Carotenoids	Quartile 1	Quartile 2		Quartile 3		Quartile 4		*P* for trend
		OR (95% CI)	*P*	OR (95% CI)	*P*	OR (95% CI)	*P*	
α-carotene								
Model 1	Reference	0.82 (0.63, 1.08)	0.1539	0.73 (0.56, 0.97)	0.0299	0.53 (0.39, 0.72)	<0.0001	<0.0001
Model 2	Reference	0.66 (0.48, 0.89)	0.0068	0.45 (0.32, 0.62)	<0.0001	0.35 (0.25, 0.50)	<0.0001	<0.0001
Model 3	Reference	0.72 (0.52, 0.99)	0.0446	0.54 (0.38, 0.78)	0.0008	0.51 (0.35, 0.76)	0.0010	0.0003
α-cryptoxanthin								
Model 1	Reference	0.57 (0.44, 0.74)	<0.0001	0.37 (0.27, 0.49)	<0.0001	0.31 (0.23, 0.43)	<0.0001	<0.0001
Model 2	Reference	0.56 (0.42, 0.75)	<0.0001	0.35 (0.25, 0.48)	<0.0001	0.32 (0.23, 0.46)	<0.0001	<0.0001
Model 3	Reference	0.61 (0.45, 0.83)	0.0015	0.44 (0.31, 0.64)	<0.0001	0.46 (0.31, 0.68)	<0.0001	<0.0001
β-carotene								
Model 1	Reference	0.92 (0.70, 1.20)	0.5351	0.66 (0.49, 0.88)	0.0046	0.65 (0.48, 0.87)	0.0035	0.0005
Model 2	Reference	0.64 (0.47, 0.87)	0.0046	0.40 (0.29, 0.55)	<0.0001	0.35 (0.25, 0.49)	<0.0001	<0.0001
Model 3	Reference	0.71 (0.51, 0.98)	0.0365	0.46 (0.32, 0.66)	<0.0001	0.53 (0.36, 0.77)	0.0010	<0.0001
β-cryptoxanthin								
Model 1	Reference	0.67 (0.51, 0.87)	0.0031	0.57 (0.43, 0.76)	<0.0001	0.42 (0.31, 0.57)	<0.0001	<0.0001
Model 2	Reference	0.62 (0.46, 0.84)	0.0019	0.57 (0.41, 0.79)	0.0007	0.39 (0.27, 0.55)	<0.0001	<0.0001
Model 3	Reference	0.70 (0.51, 0.96)	0.0290	0.65 (0.46, 0.93)	0.0174	0.58 (0.40, 0.86)	0.0061	0.0036
Lutein/zeaxanthin								
Model 1	Reference	0.72 (0.55, 0.94)	0.0152	0.58 (0.44, 0.77)	0.0001	0.46 (0.34, 0.62)	<0.0001	<0.0001
Model 2	Reference	0.70 (0.52, 0.94)	0.0179	0.51 (0.37, 0.71)	<0.0001	0.39 (0.28, 0.55)	<0.0001	<0.0001
Model 3	Reference	0.88 (0.64, 1.20)	0.4093	0.64 (0.45, 0.91)	0.0119	0.56 (0.39, 0.82)	0.0025	0.0007
Lycopene								
Model 1	Reference	0.79 (0.61, 1.03)	0.0795	0.51 (0.38, 0.69)	<0.0001	0.50 (0.37, 0.67)	<0.0001	<0.0001
Model 2	Reference	0.85 (0.64, 1.14)	0.2839	0.56 (0.40, 0.78)	0.0005	0.70 (0.50, 0.97)	0.0308	0.0031
Model 3	Reference	0.85 (0.62, 1.16)	0.2958	0.53 (0.37, 0.76)	0.0005	0.70 (0.49, 0.99)	0.0441	0.0043
Total serum carotenoids								
Model 1	Reference	0.77 (0.59, 1.00)	0.0478	0.48 (0.36, 0.64)	<0.0001	0.42 (0.31, 0.57)	<0.0001	<0.0001
Model 2	Reference	0.75 (0.56, 1.01)	0.0564	0.45 (0.33, 0.62)	<0.0001	0.36 (0.26, 0.51)	<0.0001	<0.0001
Model 3	Reference	0.82 (0.60, 1.11)	0.2003	0.51 (0.36, 0.73)	0.0002	0.52 (0.36, 0.75)	0.0005	<0.0001

Model 1: unadjusted model.

Model 2: adjusted for age, gender, ethnicity, marital status, education level, and PIR.

Model 3: additionally adjusted for the covariates in Model 2, as well as BMI, smoking, alcohol consumption, physical activity, diabetes, hypertension, CHD, stroke, thyroid problems, and cancer/malignant.

**Figure 2:**
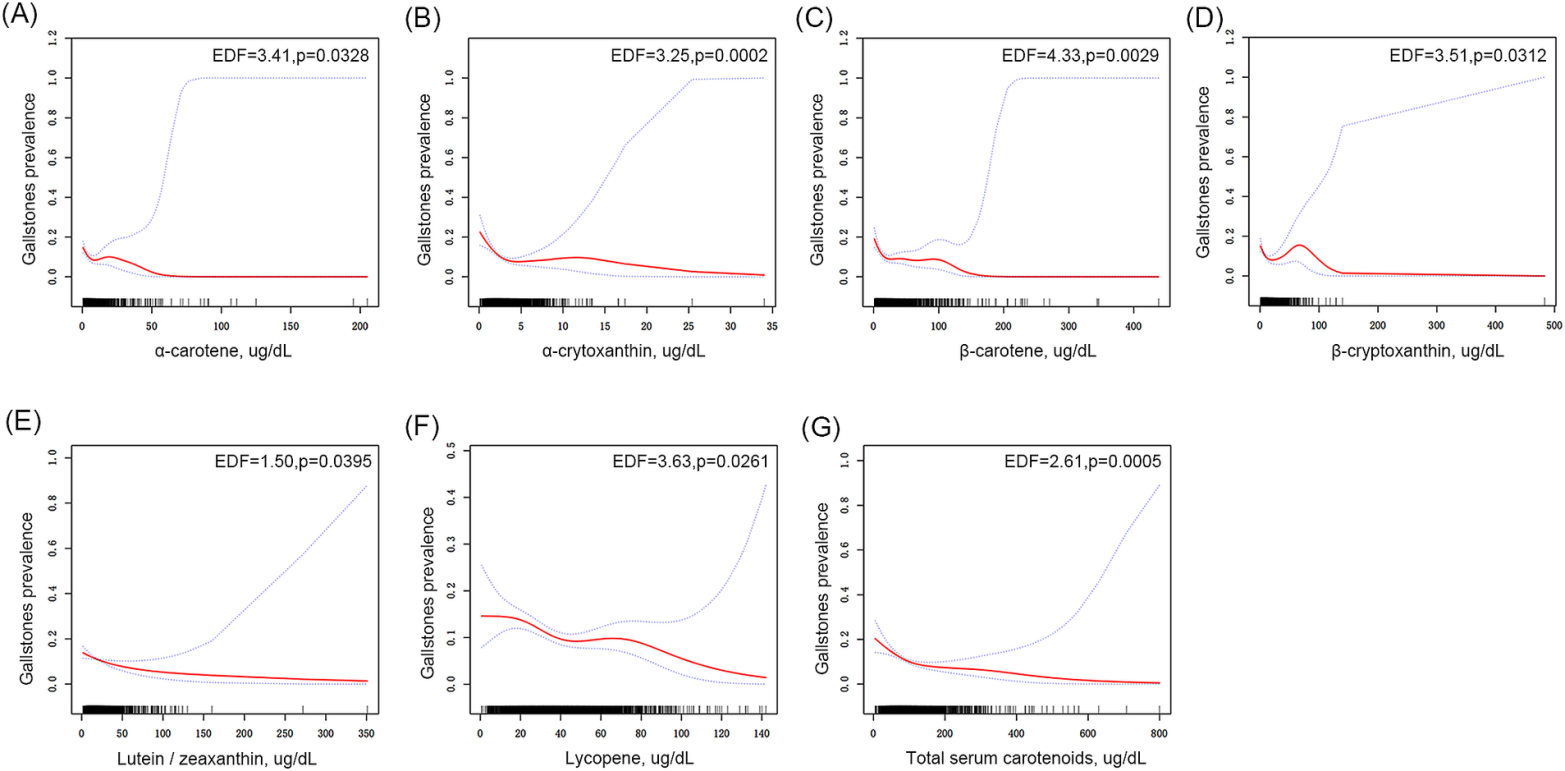
Association between serum carotenoid levels and gallstones in adults. **(A)** α-Carotene, **(B)** α-cryptoxanthin, **(C)** β-carotene, **(D)** β-cryptoxanthin, **(E)** lutein/zeaxanthin, **(F)** lycopene, and **(G)** total carotenoids in relation to gallstone prevalence. The area between the upper and lower dashed lines represents 95% confidence intervals (CI).

Subsequently, GAM regression combined with smooth curve fitting techniques were used to comprehensively explore the potential association between serum carotenoid levels and gallstones in adults (Figure 2). The results indicate a complex non-linear negative association between these carotenoids and gallstones in adults, with increased carotenoid levels potentially associated with a statistically significant reduction in gallstone prevalence.

### 3.3 Associations between the combine of six serum carotenoids and gallstones in adults

**Figure 3:**
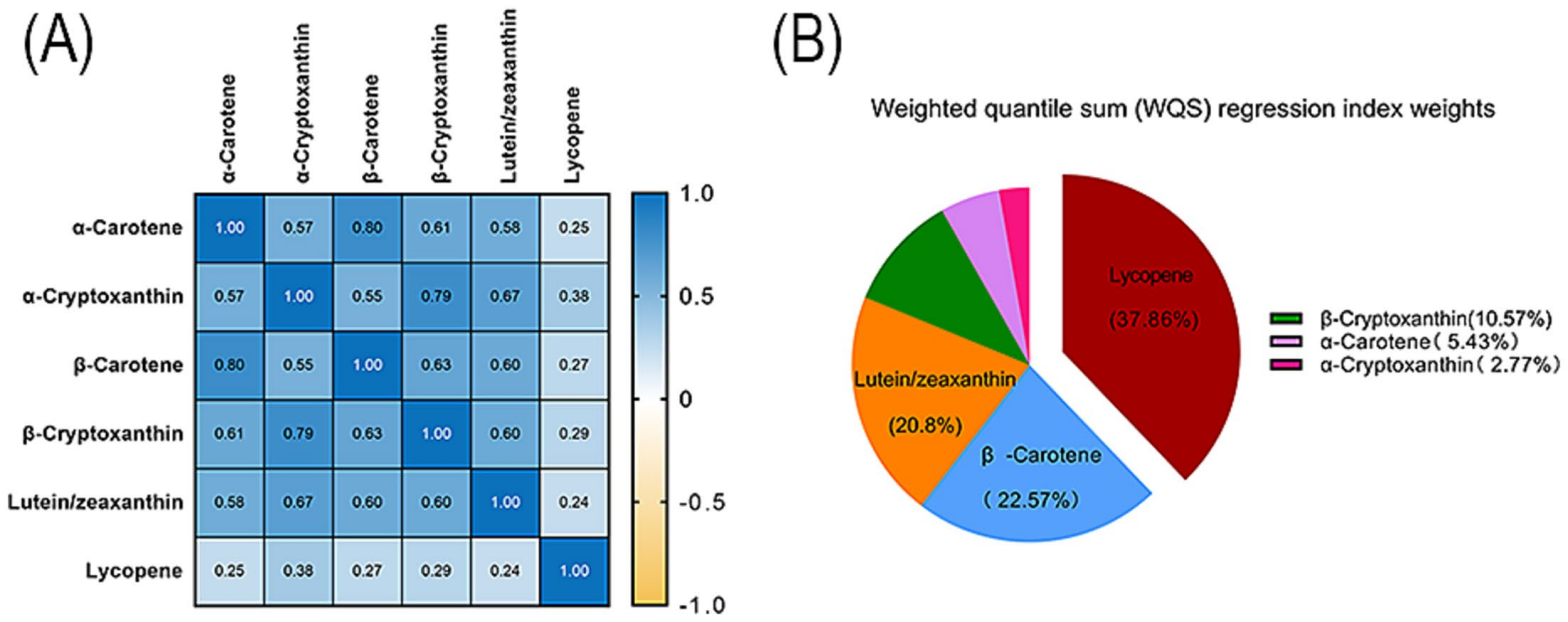
Association between the combination of the six serum carotenoids and gallstones in adults. **(A)** Pairwise Spearman correlation coefficients for serum carotenoids. **(B)** The WQS regression index weights for each carotenoid in adults with gallstones. The WQS regression was adjusted for age, sex, ethnicity, marital status, education level, PIR, BMI, smoking, alcohol consumption, physical activity, diabetes, hypertension, coronary heart disease, stroke, thyroid problems, and cancer/malignancy.

The pairwise Spearman correlation coefficients among the six serum carotenoids ranged from 0.25 to 0.80, reflecting varying degrees of inter-correlation (Figure 3A). Weighted quantile sum (WQS) regression analysis indicated that lycopene made the most substantial contribution to the association with gallstones, accounting for 37.86%, followed by β-carotene at 22.57% and lutein/zeaxanthin at 20.8% (Figure 3B). These results suggest a robust association between the levels of the three serum carotenoids and reduced gallstone prevalence.

### 3.4 Subgroup analysis and mediation analysis

This study selected the TSC level for subgroup and mediation analyses. Given the correlation between carotenoids, the use of the TSC level simplified the analytical process, allowing for a clearer identification of its association with gallstone prevalence. The results showed that, as shown in Table 4, there was a general negative association between the TSC level and gallstone prevalence. However, this correlation was not statistically significant in any subgroup. Specifically, among participants younger than 40 years, those with high school education or less, and individuals with a PIR of 1.3 or lower, the link between carotenoid levels and gallstone prevalence was not statistically significant. In contrast, within the subgroup of participants aged over 40 years, especially among those between 41 and 60 years and those over 61 years, a significant association was observed, wherein increased carotenoid levels were correlated with a reduced gallstone prevalence. Sex-specific analysis revealed that women in the third and fourth quartiles experienced a statistically significant decrease in gallstone prevalence (*p* < 0.05), whereas men demonstrated a notable reduction in gallstone prevalence in quartile three (*p* = 0.0134) and a nearly significant reduction in quartile four (*p* = 0.0547). Additionally, variables such as marital status, educational attainment, ethnicity, BMI, hypertension, and diabetes exhibited comparable trends in the specific subgroups. Overall, a consistent association emerged wherein increased serum carotenoid levels were associated with a lower gallstone prevalence across the majority of subgroups, with the exception of those participants younger than 40, possessing a high school education or lower, and having a PIR of 1.3 or less, where this association was less evident. The interaction analysis did not reveal any significant interactions (*p* > 0.05), indicating that the association between carotenoid levels and gallstone prevalence remained relatively stable across various subgroups.

**Table 4:** Subgroup analysis of the association between total serum carotenoid level and gallstones.

Characteristics	Quartile 2	Quartile 3	Quartile 4	*P* for interaction
	OR (95% CI), *p*	OR (95% CI), *p*	OR (95% CI), *p*	
Age, years				0.4744
<=40	1.70 (0.79, 3.67) 0.1786	0.63 (0.23, 1.71) 0.3620	1.22 (0.40, 3.71) 0.7242	
>40, <=60	0.52 (0.29, 0.91) 0.0218	0.49 (0.28, 0.87) 0.0153	0.39 (0.21, 0.74) 0.0037	
>60	0.84 (0.53, 1.31) 0.4369	0.49 (0.29, 0.81) 0.0059	0.46 (0.27, 0.79) 0.0052	
Gender				0.8641
Male	0.81 (0.47, 1.40) 0.4609	0.44 (0.23, 0.84) 0.0134	0.49 (0.23, 1.01) 0.0547	
Female	0.82 (0.56, 1.20) 0.3140	0.56 (0.37, 0.86) 0.0076	0.55 (0.36, 0.86) 0.0079	
Marital status				0.4059
Married/Living with Partner	0.86 (0.58, 1.28) 0.4579	0.50 (0.32, 0.79) 0.0027	0.45 (0.28, 0.73) 0.0011	
Widowed/Divorced/Separated	0.79 (0.47, 1.31) 0.3577	0.56 (0.31, 1.00) 0.0497	0.75 (0.41, 1.39) 0.3614	
Education				0.4559
<High school	1.14 (0.53, 2.49) 0.7352	0.52 (0.20, 1.35) 0.1793	0.87 (0.35, 2.16) 0.7634	
High school	1.08 (0.58, 2.00) 0.8185	0.53 (0.25, 1.10) 0.0882	0.76 (0.36, 1.61) 0.4731	
>High school	0.62 (0.41, 0.95) 0.0298	0.53 (0.34, 0.84) 0.0065	0.40 (0.24, 0.66) 0.0005	
Ethnicity				0.7553
Mexican American	1.18 (0.42, 3.29) 0.7506	0.21 (0.05, 0.82) 0.0255	0.88 (0.31, 2.50) 0.8091	
Non-Hispanic White	0.95 (0.61, 1.46) 0.8074	0.55 (0.33, 0.92) 0.0231	0.57 (0.31, 1.02) 0.0598	
Non-Hispanic Black	0.81 (0.38, 1.71) 0.5820	0.69 (0.30, 1.56) 0.3689	0.32 (0.11, 0.94) 0.0391	
Other Races	0.45 (0.21, 0.97) 0.0425	0.50 (0.24, 1.03) 0.0590	0.36 (0.16, 0.78) 0.0100	
BMI, kg/m^2^				0.9308
<=25	0.66 (0.23, 1.90) 0.4363	0.83 (0.32, 2.18) 0.7066	0.30 (0.11, 0.86) 0.0254	
>25, <=30	0.72 (0.38, 1.37) 0.3215	0.54 (0.28, 1.05) 0.0715	0.51 (0.26, 1.02) 0.0568	
>30	0.88 (0.59, 1.30) 0.5115	0.43 (0.27, 0.70) 0.0007	0.60 (0.35, 1.02) 0.0608	
PIR				0.4799
<=1.3	0.85 (0.45, 1.59) 0.6092	0.71 (0.35, 1.46) 0.3507	0.86 (0.38, 1.94) 0.7182	
>1.3, <=3.5	0.96 (0.61, 1.51) 0.8560	0.45 (0.26, 0.78) 0.0039	0.51 (0.29, 0.90) 0.0191	
>3.5	0.62 (0.33, 1.16) 0.1317	0.49 (0.26, 0.93) 0.0285	0.39 (0.20, 0.78) 0.0070	
Hypertension				0.2770
No	0.70 (0.46, 1.06) 0.0929	0.48 (0.30, 0.77) 0.0022	0.60 (0.36, 0.98) 0.0399	
Yes	0.99 (0.61, 1.59) 0.9518	0.52 (0.30, 0.90) 0.0187	0.45 (0.25, 0.80) 0.0071	
Diabetes				0.5681
No	0.79 (0.53, 1.16) 0.2260	0.58 (0.39, 0.89) 0.0114	0.43 (0.27, 0.69) 0.0004	
Yes	0.84 (0.49, 1.45) 0.5350	0.32 (0.16, 0.64) 0.0014	0.78 (0.40, 1.50) 0.4511	
CHD				0.8907
No	0.54 (0.17, 1.72) 0.3009	0.09 (0.01, 0.54) 0.0083	0.83 (0.17, 3.95) 0.8137	
Yes	0.83 (0.60, 1.15) 0.2549	0.57 (0.40, 0.82) 0.0023	0.53 (0.36, 0.78) 0.0014	

BMI, body mass index; PIR, poverty income ratio; CHD, coronary heart disease.

**Figure 4:**
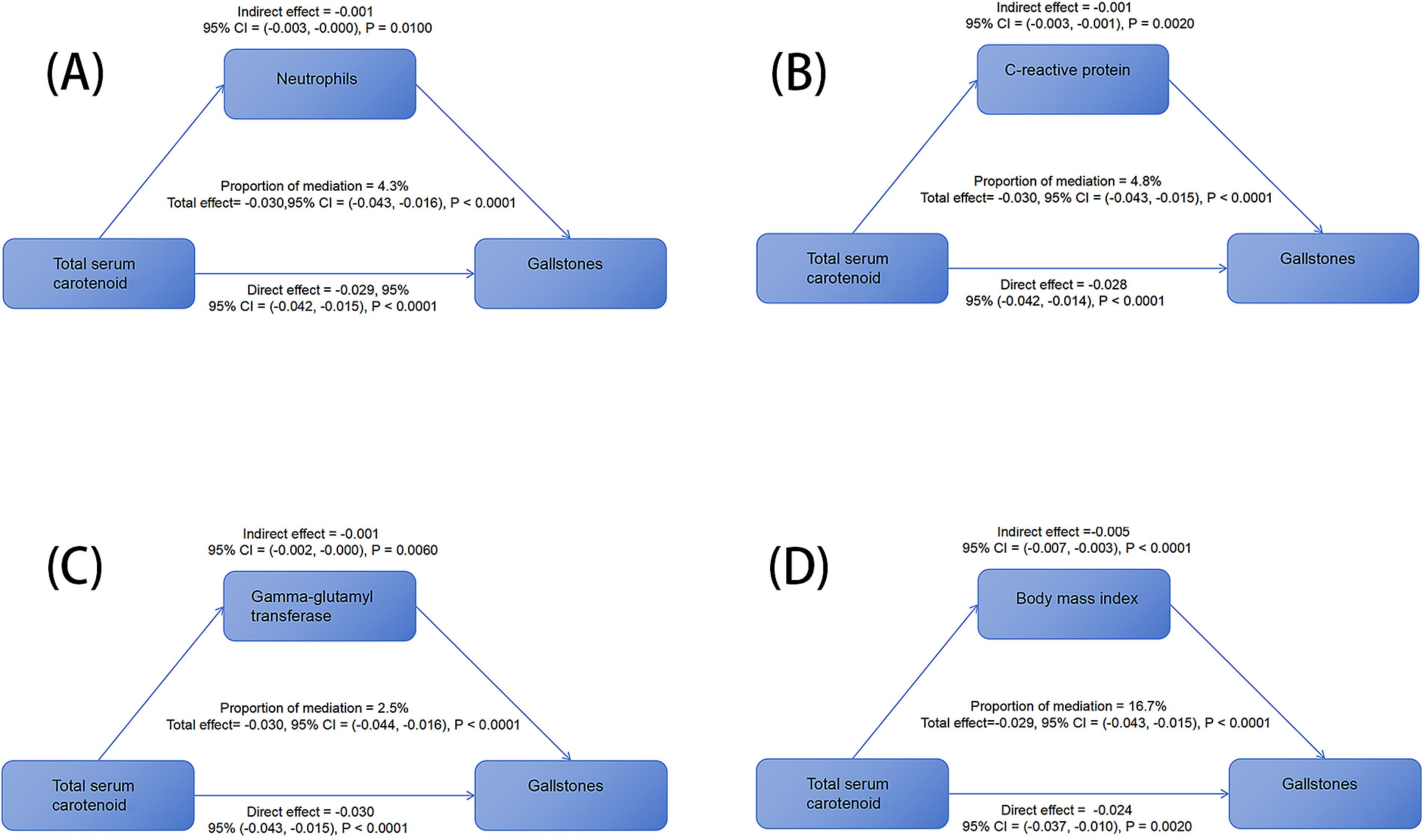
Different mediating variables on the association between total serum carotenoid level and gallstone prevalence. **(A)** Neutrophil count, **(B)** C-reactive protein, **(C)** gamma-glutamyl transferase, and **(D)** BMI in mediation analysis of the association between total serum carotenoid level and gallstone prevalence.

Mediation analysis was performed to assess the influence of different mediating variables on the association between the TSC level and gallstone prevalence. The models and pathways used for the mediation analysis are depicted in Figure 4. After controlling for potential confounding variables, the findings revealed that although the indirect effects of most indicators were not significant, the indirect effects related to neutrophil count (−0.001, *p* = 0.0100), CRP (−0.001, *p* = 0.0020), and GGT (−0.001, *p* = 0.0600) were statistically significant. Furthermore, the total effect of BMI was calculated at −0.029, with an indirect effect of −0.005 and a direct effect of −0.024, yielding a mediation proportion of 16.7%. This result highlights the potential impact of BMI on this association; although the mediation proportion of 16.7% is not particularly high, it is considered significant in this study. In summary, carotenoids may indirectly mitigate the gallstone risk by affecting variables such as obesity and inflammation while potentially providing a direct protective effect. Table 5 summarizes the mediation analysis results, encompassing direct effects, indirect effects, total effects, and mediation ratios.

**Table 5:** Mediation analysis of liver function, uric acid, inflammatory markers, obesity, blood lipids, diabetes, and insulin resistance in the association between total serum carotenoid level and gallstones.

Characteristics	Mediation effect (95% CI), *p*-value			
	Total effect	Indirect effect	Direct effect	Mediation
TG	−0.023 (−0.041, −0.003) 0.0140	−0.000 (−0.001, 0.001) 0.9520	−0.023 (−0.041, −0.004) 0.0140	0%
TC	−0.031 (−0.045, −0.017) < 0.0001	0.000 (−0.004, 0.005) 0.8500	−0.032 (−0.047, −0.017) < 0.0001	1.2%
WBC	−0.030 (−0.044, −0.017) < 0.0001	−0.001 (−0.002, 0.000) 0.1000	−0.030 (−0.043, −0.015) < 0.0001	2.4%
NEU	−0.030 (−0.043, −0.016) < 0.0001	−0.001 (−0.003, −0.000) 0.0100	−0.029 (−0.042, −0.015) < 0.0001	4.3%
HbA1c	−0.030 (−0.044, −0.017) < 0.0001	0.000 (−0.000, 0.000) 0.9620	−0.030 (−0.044, −0.017) < 0.0001	0%
CRP	−0.030 (−0.043, −0.015) < 0.0001	−0.001 (−0.003, −0.001) 0.0020	−0.028 (−0.042, −0.014) < 0.0001	4.8%
FI	−0.023 (−0.041, −0.004) 0.0140	−0.001 (−0.003, 0.000) 0.0520	−0.022 (−0.040, −0.002) 0.0160	4.7%
FER	−0.031 (−0.044, −0.016) < 0.0001	−0.000 (−0.001, 0.000) 0.4820	−0.031 (−0.044, −0.016) < 0.0001	0.2%
FBG	−0.023 (−0.042, −0.004) 0.0180	0.000 (−0.000, 0.001) 0.7640	−0.023 (−0.042, −0.004) 0.0160	0.1%
ALT	−0.030 (−0.044, −0.016) < 0.0001	−0.000 (−0.001, 0.000) 0.0600	−0.030 (−0.043, −0.016) < 0.0001	1.4%
AST	−0.030 (−0.044, −0.016) < 0.0001	−0.000 (−0.001, 0.000) 0.3240	−0.030 (−0.044, −0.016) < 0.0001	0.5%
GGT	−0.030 (−0.044, −0.016) < 0.0001	−0.001 (−0.002, −0.000) 0.0060	−0.030 (−0.043, −0.015) < 0.0001	2.5%
LDH	−0.031 (−0.045, −0.016) < 0.0001	0.000 (−0.000, 0.000) 0.9660	−0.031 (−0.045, −0.017) < 0.0001	0%
TB	−0.030 (−0.044, −0.016) < 0.0001	−0.000 (−0.001, 0.000) 0.2780	−0.030 (−0.043, −0.016) < 0.0001	0.9%
UA	−0.030 (−0.043, −0.016) < 0.0001	−0.001 (−0.003, −0.000) 0.0220	−0.029 (−0.042, −0.014) < 0.0001	4.3%
BMI	−0.029 (−0.043, −0.015) < 0.0001	−0.005 (−0.007, −0.003) < 0.0001	−0.024 (−0.037, −0.010) 0.0020	16.7%

Adjust for: age, gender, ethnicity, marital status, education level, PIR, smoking, alcohol consumption, physical activity, diabetes, hypertension, CHD, stroke, thyroid problems, and cancer/malignant.

## 4 Discussion

This study used data from the 2017–2018 National Health and Nutrition Examination Survey (NHANES) to investigate the association between serum carotenoid levels and the occurrence of gallstones in adult populations. These findings revealed a significant inverse association between serum carotenoid levels and the likelihood of gallstone development. Individuals in the highest quartile of total carotenoid levels exhibited a 48% lower risk of gallstone formation than those in the lowest quartile. Similar trends are observed for specific carotenoids: *α*-carotene is associated with a 49% decrease in risk (Q4: OR = 0.51, *p* = 0.0010), α-cryptoxanthin with a 54% decrease (Q4: OR = 0.46, *p* < 0.0001), β-carotene with a 47% reduction (Q4: OR = 0.53, *p* = 0.0010), β-cryptoxanthin with a 42% reduction (Q4: OR = 0.58, *p* = 0.0061), lutein/zeaxanthin with a 44% reduction (Q4: OR = 0.56, *p* = 0.0025), and lycopene with a 30% reduction (Q4: OR = 0.70, *p* = 0.0441). These results imply that elevated serum carotenoid levels may confer protective effects against gallstone development.

Serum carotenoid levels not only reflect an individual’s dietary habits but are also associated with various health conditions. Research has found an inverse correlation between serum carotenoid concentrations and the risk of several chronic diseases, including mortality rates in patients with metabolic syndrome, endometriosis, osteoarthritis, cardiovascular-renal-metabolic syndrome, suicidal thoughts, adult migraines, hyperuricemia, and so on (28, 32–35). While certain studies have examined the relationships among oxidative balance scores, dietary quality metrics, and gallstones, potentially considering the dietary intake of carotenoids (5, 31), these metrics are distinct from serum carotenoid levels, which more accurately reflect the actual bioavailability and metabolic activity. The use of serum carotenoid levels reduced the likelihood of recall bias and inaccuracies. To our knowledge, this is the first study to evaluate the association between serum carotenoid levels and gallstones in adult cohorts using a large sample size.

Subgroup analyses reveal that the inverse association between serum carotenoid levels and gallstone risk remains consistent across various demographics, with the exception of individuals younger than 40 years, those with a high school education or lower, and those with a PIR of ≤1.3, where this correlation is less evident. This variation may be due to multiple factors. First, metabolic characteristics, inflammatory responses, and oxidative stress levels can differ markedly between younger and older populations (36). Gallbladder function and bile metabolism in individuals aged <40 years may exhibit reduced sensitivity to the protective effects of carotenoids. Second, socioeconomic factors may exert a considerable influence; individuals with lower educational and income levels may experience heightened nutritional deficiencies and lifestyle challenges, which could affect their dietary patterns, antioxidant capacity, and overall health (37), thereby affecting carotenoid bioavailability (38).

This study identified potential mediating factors in the association between serum carotenoid levels and gallstone formation through mediation analysis. The mediating roles of neutrophil count, CRP, and GGT highlighted the significance of inflammation and liver function in this association. Previous research has found that carotenoid supplementation significantly reduces levels of CRP, IL-6, and TNF-α, with β-carotene and lutein/zeaxanthin showing particularly pronounced effects on lowering CRP levels (39). Additionally, carotenoid oxidation products generated after oxidative stress may induce neutrophil apoptosis, potentially shortening the duration of inflammation (40). Furthermore, carotenoids may protect liver function by enhancing antioxidant capacity and inhibiting GGT elevation (41). These findings provide strong explanations for the results. Moreover, previous studies have reported a negative association between serum carotenoid levels and the prevalence of metabolic disorders, such as obesity, overweight, and lipid metabolism disorders (42). Our study demonstrated that BMI played a pivotal, partially mediating role in the association between carotenoid levels and gallstone formation, with a significant negative mediating effect. This indicates that the effect of serum carotenoid levels on gallstone formation is likely to a large extent driven by their influence on obesity. Specifically, higher serum carotenoid levels are associated with lower BMI, which in turn may reduce the risk of gallstone formation. This finding is consistent with previous research characterizing obesity as chronic low-grade inflammation and increased oxidative stress, suggesting that serum carotenoid levels may indirectly regulate the risk of gallstone formation by affecting BMI (43). The fact that BMI partially mediated this association further underscores the importance of weight management for the favorable effects of serum carotenoid levels on gallstones.

The strengths of this study are as follows: First, based on NHANES data, it included 3,809 adults aged 20 and older, ensuring a large sample size. The stratified, multistage sampling design guarantees broad representativeness of the sample. Covariates were assessed using standardized questionnaires, minimizing measurement bias, and enabling the findings to be widely applied to the adult population in the United States. Second, multiple statistical methods were employed, including multivariate logistic regression, GAM with smoothing curve-fitting techniques, and subgroup analyses. These methods have examined the association between serum carotenoid levels and gallstones from various perspectives. The mediation analysis also provided new insights into the potential mechanisms underlying the effects of carotenoids on gallstone risk. Third, serum carotenoid levels were directly measured rather than relying on dietary recall, avoiding recall bias and allowing for a more accurate assessment. However, this study had several limitations. The cross-sectional nature of this study limited causal inferences. Relying on self-reported data for gallstone diagnosis may introduce bias owing to subjective recall errors, underreporting of asymptomatic cases, or misinterpretation of symptoms (e.g., mistaking other digestive disorders for gallstones), thereby impacting the validity of the results. Although this study highlighted the potential protective effects of serum carotenoids, it is important to consider the effects of dietary sources and lifestyle factors on carotenoid levels and gallstone risk. Variations in dietary habits, physical activity levels, and overall nutritional health can significantly influence carotenoid bioavailability and efficacy, thereby affecting the interpretation of results. Additionally, the NHANES data primarily reflect the adult population of the United States, limiting the extrapolation of findings to regions with significantly different dietary patterns or genetic susceptibilities related to bile metabolism. Finally, because gallstone formation is a long-term process, a single measurement of serum carotenoid levels at a single time point may not fully capture the effects of long-term exposure.

In this study, to ensure methodological validity, we used NHANES stratified sampling to represent the United States adult population and excluded irrelevant subgroups (individuals aged <20 years and those with missing data) to minimize selection bias. Our statistical models underwent rigorous validation: stepwise-adjusted multivariate regression (models 1–3) isolated carotenoids’ independent effects; GAM regression combined with smooth curve fitting confirmed non-linear associations (EDF > 1, *p* < 0.05); WQS regression identified key components (lycopene, β-carotene, and lutein/zeaxanthin). The consistent inverse associations across carotenoid subtypes (OR: 0.46–0.70) and most subgroups (except young/low-education/low-income ones) further strengthened the results’ reliability. Acknowledging the limitations of the cross-sectional design in terms of causal inference, we interpreted the data with caution. However, our findings align with the existing literature on the antioxidant and anti-inflammatory properties of carotenoids and their established benefits in reducing various chronic disease risks, supporting the plausibility of our results. Mediation analysis also indicated that BMI played a significant intermediary role, suggesting that the protective effect of carotenoids on gallstone risk might partly result from their impact on body weight. Overall, our comprehensive evaluation of the methods, results, and data interpretation indicates this study offers valuable insights into the potential protective link between serum carotenoid levels and gallstone risk, suggesting that maintaining higher serum carotenoid levels may reduce the risk of developing gallstones. For populations at a high gallstone risk, such as those with obesity, dietary interventions or dietary supplements to increase serum carotenoid levels may help reduce the gallstone risk. Future research should further explore the specific mechanisms by which carotenoids act as protective factors against gallstones and validate the causal relationships through longitudinal studies to provide a more targeted theoretical basis for the prevention and management of gallstones.

## 5 Conclusion

This cross-sectional analysis based on NHANES data involving 3,809 adults showed a significant negative association between serum carotenoid levels and gallstone risk. The highest quartile of total carotenoids was associated with a 48% lower gallstone risk than the lowest quartile. Six specific carotenoids, including *α*-carotene and α-cryptoxanthin, exhibited dose-dependent protective effects, and GAM analysis further confirmed the non-linear inverse association between them. Subgroup analyses yielded robust results, and mediation analysis indicated that BMI mediated 16.7% of the total effect. This study is the first to demonstrate a negative association between serum carotenoid levels and gallstone occurrence in adults using large-sample data. Although a cross-sectional design cannot establish causality, the results suggest that maintaining high serum carotenoid levels may reduce the gallstone risk. For populations at a high gallstone risk, such as those with obesity, dietary interventions or dietary supplements to increase serum carotenoid levels may help reduce the gallstone risk. Future research is needed to further elucidate the protective mechanisms of serum carotenoids and validate causal relationships through longitudinal studies, thus laying a solid scientific foundation for the prevention and management of gallstones.

## Data Availability

The datasets presented in this study can be found in online repositories. The names of the repository/repositories and accession number(s) can be found in the article/supplementary material.

## Footnotes

^1^ http://www.R-project.org

^2^ http://www.empowerstats.com
